# Low Vitamin B12 Levels: An Underestimated Cause Of Minimal Cognitive Impairment And Dementia

**DOI:** 10.7759/cureus.6976

**Published:** 2020-02-13

**Authors:** Shazia Jatoi, Abdul Hafeez, Syeda Urooj Riaz, Aijaz Ali, Muhammad Ishaq Ghauri, Maham Zehra

**Affiliations:** 1 Medicine, Jinnah Medical and Dental College, Karachi, PAK; 2 Neurology, Liaquat University of Medical and Health Sciences, Liaquat University Hospital Jamshoro, Hyderabad, PAK; 3 Internal Medicine, Jinnah Medical College Hospital, Karachi, PAK

**Keywords:** vitamin b12 deficiency, acetyl homocysteine, cognition, memory deficit, mental health

## Abstract

Background

Vitamin B12 deficiency is linked to impaired cognition and memory along with a sensation of tingling and numbness, an outcome of poor myelination. Elevated methylmalonic acid and serum homocysteine levels are markers of Vitamin B12 deficiency. Elevated homocysteine levels are also often associated with Alzheimer’s disease and stroke. We conducted this study to determine the effect of vitamin B12 replacement therapy on vitamin B12-deficient patients with noted cognitive impairment.

Methods

We conducted a cross-sectional, multicenter study of patients with minimal cognitive impairment (MCI) to assess for Vitamin B12 and homocysteine levels. All patients found to be deficient in vitamin B12 underwent replacement therapy and were assessed again after three months via the Mini-Mental State Examination (MMSE) and a review of symptoms.

Results

A total of 202 patients were included in the study. Of those, 171 (84%) patients reported marked symptomatic improvement after vitamin B12 replacement while MMSE scores improved in 158 (78%) patients. Of the remaining 44 patients who reported no symptomatic improvement, MMSE scores improved in 26 while 18 patients showed no MMSE score improvements.

Conclusions

Vitamin B12 deficiency is linked to cognition, and replacement therapy may be an option to improve patient cognition outcomes. Further studies are needed to confirm and refine the observed associations over a larger scale and to determine whether these findings will translate to a reduction in cognitive decline.

## Introduction

Vitamin deficiencies have long been known to cause various effects on human health. Vitamin B12 deficiency has been correlated to various neurologic problems; however, research has suggested that abnormally low levels of vitamin B12 can be the cause of significant cognitive dysfunction [1]. Low vitamin B12 levels can cause serious health issues (e.g., megaloblastic anemia, inhibition of cell division, and dysfunctional myelination). Elevated methylmalonic acid and serum homocysteine levels are specific markers of vitamin B12 deficiency, and are also associated with poor myelination. Elevated homocysteine levels may be associated with Alzheimer’s disease, cardiovascular disease, and stroke [2-4]. Low vitamin B12 and high homocysteine levels may cause silent brain injury through oxidative stress, resulting in calcium influx and apoptosis or from the formation of oxidized homocysteine (e.g., homocysteine sulfinic acid and homocysteine acid) [5-6].

In general, vitamin B12 levels decline with age, and therefore, the prevalence of vitamin B12 deficiency increases in the elderly population [7]. As noted by Wong, there is no precise definition or gold standard test to diagnose vitamin B12 deficiency; rather, the diagnosis is usually based on identifying a low level of serum vitamin B12 along with the clinical evidence of deficiency, which response to replacement therapy [8]. The initial laboratory assessment includes serum vitamin B12 level, complete blood count, and blood film along with additional information about serum homocysteine levels [8]. There is no universally accepted cutoff value to define the deficiency, but a value of less than 203 pg/mL is considered low. The World Health Organization has suggested the use of this cutoff value since 2008; however, as it has been observed that neurologic manifestations appear at levels above this cutoff value (probably because of poor myelination), a higher cutoff value of 298-350 pg/mL has been suggested [9-10].

Dietary deficiency of vitamin B12 plays a major role in the causation of a state of clinical deficiency. In the developing world, poor socioeconomic status plays a major role in inadequate levels of nutrition. In young adults, a major cause of vitamin deficiency is the inadequate consumption of animal-source food. In older patients, malabsorption may be the main cause of the deficient state [11]. A study conducted in South India indicated that the prevalence of absolute deficiency was 14.9%, while 37.6% had borderline deficiency [12]. A similar study conducted in Pakistan in a hypothyroid population showed that almost 24% of the patients between the age of 30 and 70 years were deficient in vitamin B12 [13].

However, in developed countries, the causes of vitamin deficiency vary beyond consumption. A French study showed that among 172 patients with vitamin B12 deficiency, only 2% accounted for an inadequate dietary intake; therefore, the deficiency arises from other factors such as malabsorption, postgastrectomy state, bacterial overgrowth, and long-term use of medications for some comorbidities (e.g., proton pump inhibitors and H2 receptor-blocking drugs, which might interfere with the release of vitamin B12 from food sources) [14].

In Pakistan, no such study had been conducted on the causes and effects of vitamin B12 deficiency in general, especially through the lens of mental health. We conducted this study in a low socioeconomic area of Karachi in patients attending a neurology outpatient department with a variety of neurologic manifestations.

According to Lavretsky, minimal cognitive impairment (MCI) is defined as a cognitive decline that is greater than expected for an individual’s age and educational level but that does not interfere with the activities of daily living [15]. It can involve problems with memory, language, thinking, and judgment [16]. Our current study was conducted to correlate any causative association between serum vitamin B12 levels and MCI and to see if the supplementation improved the outcome.

## Materials and methods

This study was a cross-sectional, multicenter project conducted at Jinnah Medical College Hospital in Korangi and Medicare Cardiac and General Hospital in Karachi. Patients at these two centers who presented in the neurology outpatient department from January 2017 to December 2017 and provided written informed consent were selected for inclusion. 

Although vitamin B12-deficient patients have a wide variety of vague neurologic manifestations, our present study was specifically focused on cognitive impairment. All patients above the age of 18 years who presented with concerns of forgetfulness, poor focus and concentration, memory decline, generalized tiredness, lethargy, paresthesias, and tingling sensation, and poor balance were included in this study. As our main focus in this study was on MCI, we assessed patients for clinical signs of MCI and used the Mini-Mental State Examination (MMSE), Backward Digit Span test (from the revised Wechsler Adult Intelligence Scale), and the East Boston Memory Test (EBMT).

All patients who fulfilled the clinical criteria of MCI were further assessed with a detailed neurologic examination. Patients with any focal neurologic deficit were sent for a preliminary brain imaging study (i.e., magnetic resonance imaging [MRI] of the brain with or without contrast, depending on the expected lesion). Finally, only those patients were selected for this study who had MCI but for whom no focal cause was found.

All selected patients were further assessed for any metabolic cause of memory impairment by blood sampling. Patients with no other major metabolic derangement except for having low vitamin B12 levels or high serum homocysteine levels were considered for inclusion.

Biochemical sampling

Blood samples from the selected patients were taken and analyzed for complete blood picture, urea, creatinine, serum electrolytes, blood glucose levels, liver function tests, thyroid hormone levels, and serum vitamin B12 and homocysteine levels.

Patients with MCI and low serum levels of vitamin B12 without any evidence of focal brain lesion or any major metabolic derangement and who had gradual development of cognitive impairment without any premorbid history of similar complaints were ultimately selected for inclusion into the study.

This population of patients was offered vitamin B12 replacement therapy and was kept on a three-month follow-up. The mode of administration was planned according to the deficiency state (i.e., parenteral route was planned for those who had a severe deficiency and oral replacement for patients with mild to moderate deficiency). These patients were reassessed clinically and by biochemical analysis of vitamin B12 and homocysteine levels after six months.

## Results

Almost 8000 patients visited the neurology outpatient department over 12 months, among whom 1470 (18.3%) patients presented with the relevant concerns of poor focus, gradual memory decline or recent forgetfulness, lethargy without weight loss, and sensory paresthesias. Selected patients were older than 18 years, and most patients were older than the age of 50 years. These patients were further assessed with a detailed history, clinical examination, MMSE, EBMT, and backward counting tests. Only 281 (19%) of 1470 patients were selected to be included in this study, as these patients fulfilled the clinical criteria of MCI and had an MMSE score below 24. The lowest MMSE score in this population was 12, which was considered in the dementia category.

Among these 281 patients, 19 had a focal neurologic deficit. After neuroimaging (i.e., brain MRI), 14 patients were found to have a stroke, while five had a space-occupying lesion. Among these five patients, three had a brain tuberculoma and two were finally diagnosed to have brain tumors. These 19 patients were not included in this study. Thirteen more patients were excluded from the study group because of being diagnosed as metabolic derangements (chronic kidney disease in five and chronic liver disease in eight cases).

Finally, 249 (16.9%) of 1470 patients were selected to participate in this study, as these patients had MCI, but no discernable focal or metabolic cause for their condition. Among this patient population, 202 (13.7%) of 1470 patients had a low or lower normal range of vitamin B12 levels with elevated homocysteine levels.

Of these 202 patients, 126 were women and 76 were men. Considering the age range, 137 of 202 were older than age 55 years, 47 patients were aged between 30 and 55 years, and 18 patients were aged between 18 and 30 years.

Among these patients, 58 (28.7%) of 202 had vitamin B12 levels between 200 and 350 pg/mL and were considered to be mildly deficient, while 113 (56%) of 202 patients had a level between 100 and 200 pg/mL (moderate deficiency); 31 (15.3%) of 202 had a vitamin B12 level between 50 and 100 (severe deficiency). The minimum vitamin B12 level was 51 pg/mL.

Forty-three patients had a serum homocysteine level above 20 µmol/L, 101 patients had levels between 15 and 20 µmol/L, and 58 patients had levels between 8 and 15 µmol/L.

All the vitamin B12-deficient patients were offered vitamin B12 replacement therapy by parenteral route initially to make sure that there was no issue with absorption, followed by oral replacement therapy for three months.

These patients were reassessed after three months of therapy by detailed history and the same cognitive assessments, as well as by a repeat vitamin B12 level assessment. All the patients had vitamin B12 levels between 400 and 800 pg/mL. A total of 171 (84%) of 202 patients reported marked symptomatic improvement, while MMSE results were improved in 158 (78%) of 202 patients. Of the remaining 31 patients who reported no symptomatic improvement, MMSE outcomes were improved in 13, while 18 patients did not show any improvement in MMSE.

These 18 patients (all women over the age of 50 years) were further assessed for any other cause of symptoms (e.g., depression, anemia, thyroid dysfunction, or any anxiety disorder) and were continued on a low-dose oral supplement of vitamin B12. All results are shown in Table 1.

 

**Table 1:** Demographics and outcomes (n=202)

Variables	Frequency	Percentage
Male	76	37.6%
Female	126	62.4%
Age >55 years	137	67.8%
Age>30<55 years	47	23.3%
Adverse events >18<30	18	8.9%
Vitamin B12 mild deficiency (200-350 ng/ml)	58	28.7%
Vitamin B12 moderate deficiency (100-200 ng/ml)	113	56%
Vitamin B12 severe deficiency (50-100 ng/ml)	31	15.3%
Homocysteine levels >20 µmol/L	43	21.3%
Homocysteine levels >15<20 µmol/L	101	50%
Homocysteine levels >8<15 µmol/L	58	28.7%
Improvement in symptoms and Mini-Mental State Examination	158	78%
Improvement in Mini-Mental State Examination but not in symptoms	26	13.1%
No improvement	18	8.9%

## Discussion

The precise definition of MCI has long been controversial, but a diagnosis of MCI follows similar methods that are used to diagnose clinical dementia and Alzheimer’s disease. An algorithm assists clinicians in identifying the subjects and classifying them into various subtypes of MCI [17]. A key symposium was held in Stockholm, Sweden, in 2003, to integrate clinical and epidemiological perspectives on the topic of MCI. The specific recommendation for the general MCI criteria is the patient is neither normal nor demented; there is evidence of cognitive deterioration shown by either objectively measured decline over time or subjectively reported decline of cognition, focus, and memory by self and/or informant in conjunction with objective cognitive deficit; and activities of daily living are preserved, and complex instrumental functions are either intact or minimally impaired [18].

The long-running lack of a gold standard value denoting vitamin B12 deficiency presented a challenge to investigators, but a cut-off of 203 pg/mL was finally set, and neurologic manifestations become apparent between 298 pg/ml and 350pg/mL [9-10, 19]. Clinically significant vitamin B12 deficiency signs and symptoms (e.g., anemia, numbness and paresthesias, vertigo, ataxia, and forgetfulness) can occur in the elderly at serum vitamin B12 concentration below this cut-off value; however, a strong association was found between low normal scores of vitamin B12 and cognitive impairment in a study published by the American Society of Nutrition, and the results of our current study highly endorse the same idea [20].

In our studied population, 202 (13%) patients were found to be in a vitamin B12-deficient state; 31 (15%) of them had an absolute deficiency state with serum B12 levels between 50 pg/mL and 100 pg/mL. This population presented with significant symptoms of memory worsening, poor focus and concentration, and lethargy affecting their activities of daily living. Most of these patients had a persistent need for a caregiver to maintain their balance and carry out their routines. When replacement therapy was given in these patients, almost all of them showed a correction of serum biomarkers, but only 13 patients showed improvement of MMSE outcomes, and 18 showed no improvement in their signs and symptoms. Among the patients with serum vitamin B12 levels above 100 pg/mL, 171 (84%) had significant symptomatic improvement and 158 patients showed improvement in their MMSE score. This finding can be the basis of a hypothesis that chronic, very low levels of vitamin B12 could be the cause of permanent, or at least refractory, changes in cognition and memory, which can lead to dementia.

Research conducted in 2016 revealed that metabolic vitamin B12 deficiency was present in 10% to 40% of the population and is frequently missed, this could be easily treated if recognized early, and it may be an important opportunity to prevent dementia and stroke [21]. Elevated homocysteine levels are a risk factor for cognitive decline. A recent study found a clear association between homocysteine and the Stroop test (i.e., a measure of executive functions and cognitive flexibility but not with simpler measures of verbal memory or MMSE) [22]. Patients with Alzheimer’s disease have been found to have lower vitamin B12 levels and higher homocysteine levels as compared to patients without dementia [23]. Considering serum homocysteine levels in our patients, 43 patients had serum homocysteine levels above 20 µmol/L, 101 patients had levels between 15 and 20 µmol/L, and 58 had levels between 8 and 15 µmol/L. These high levels of homocysteine can be an independent cause of damage to neurons and can be a leading cause of dementia [24].

Given our study’s cross-sectional design, a direct association between low vitamin B12 level and cognitive impairment reflects the adverse effect of this common nutritional deficiency on mental health in all age groups. Although vitamin B12 deficiency is prevalent in elderly populations, a significant number of patients in our study were of a comparatively younger age group. Possible causes of such a common deficiency state could be gastritis-associated poor absorption, excessive use of other drugs (e.g., proton pump inhibitors), pernicious anemia, or nutritional deficiency due to poor socioeconomic status. Signs and symptoms of this deficiency are usually vague and are easily missed. The effects of treatment have shown that early identification and replacement therapy causes a significant reversal of symptoms, which is an important possible step toward a healthy mental state. As Wong reported, classical treatment for vitamin B12 deficiency is parenteral administration, usually an intramuscular injection to rebuild the tissue stores [8]. It is believed that vitamin B12 in the form of hydroxocobalamin is converted to active enzyme more easily and retained in the body for longer compared to cyanocobalamin. Generally, 1 mg daily for one week or 1 mg three times per week for two weeks followed by 1 mg per week for one month, then 1 mg per month as a maintenance dose is a recommended schedule, but the regimen varies across countries and between individual practices [8,25]. Correction of risk factors associated with vitamin B12 deficiency, such as overuse of antibiotics and proton pump inhibitors for H. pylori infection, and awareness regarding healthy and clean eating, are also important in the prevention and management of this prevalent state [8].

The strength of our study was clear control over key confounders, exclusion of other possible causes of worsening cognition to the best of our ability, and results that supported our idea of improved cognition with replacement therapy. While some data were self-reported (e.g., residual symptoms), these were not proven according to our selected scales. Our main challenges were the lack of a gold standard indicator of low vitamin B12 level causing cognitive decline and any gold standard test of MCI; however, the strong association found between low MMSE scores and low vitamin B12 status attests to its ability to capture the cognitive impairment due to this cause. This study was not carried out on a large scale; however, considering that a significant percentage of the population suffers from such a nutritional deficiency that can affect the quality of life but can be easily corrected, there is a need for a similar study to be conducted in a larger population.

## Conclusions

We found a clear association between low vitamin B12 levels and progressive cognitive impairment; therefore, further studies are needed to confirm and refine the observed associations over a larger scale and to determine whether this change will translate to a reduction in cognitive decline. Moreover, we are in favor of screening for vitamin B12 deficiency, at least in the elderly population, to find and prevent the possible causes of this deficiency state, as this can be an easily preventable cause of impending dementia before the cognitive decline becomes irreversible.
